# The many roles of C1q

**DOI:** 10.7554/eLife.61599

**Published:** 2020-09-14

**Authors:** Mark Noble, Christoph Pröschel

**Affiliations:** Department of Biomedical Genetics, University of Rochester School of Medicine and DentistryRochesterUnited States

**Keywords:** complement C1q, neural stem cell transplant, spinal cord injury, neuroepithelial stem cells, Mouse

## Abstract

The ability of a well-known component of the complement cascade to bind to a variety of receptors has implications for signaling biology, spinal cord injury and, possibly, the evolution of the complement system.

**Related research article** Benavente F, Piltti K, Hooshmand MJ, Nava AA, Lakatos A, Feld BG, Creasman D, Gershon PD, Anderson A. 2020. Novel C1q receptor-mediated signaling controls neural stem cell behavior and neurorepair. *eLife* **9**:e55732. doi: 10.7554/eLife.55732

The protein C1q is best known for its role in the immune system: as a part of the C1 complex of the complement cascade, it helps to clear microbes and cellular debris from the body. However, it also has other roles beyond the immune system: for example, working with other elements of the complement cascade it helps to eliminate immature synapses during development of the visual system (Stevens et al., 2007) and is involved in Wnt signaling in muscle cells (Naito et al., 2012). C1q can also bind to myelin-associated glycoprotein and mask its ability to inhibit neurite outgrowth (Peterson et al., 2015), while the loss of C1q in mice results in cognitive decline (Stephan et al., 2013). More recent studies suggest a role for C1q in neurodegeneration (reviewed in Cho, 2019).

Now, in eLife, Francisca Benavente (University of California Irvine and Universidad del Desarrollo), Aileen Anderson (UC Irvine) and colleagues report new roles for C1q that have intriguing implications for signaling biology, recovery from spinal cord injury, stem cell-based therapies and, possibly, the evolution of the complement system (Benavente et al., 2020). The work shows that C1q is a ligand that can bind directly to several previously unidentified receptors (CD44, GPR62, BAI1, c-MET, and ADCY5), triggering the activation of downstream signaling pathways. The researchers demonstrate that in doing so, C1q can modulate different aspects of the biology of neuroepithelial stem cells (NSCs). Specifically, they show that C1q promotes the migration of NSCs by binding to a cell-surface glycoprotein called CD44, while promoting NSC proliferation via interactions with G-protein coupled receptor signaling pathways.

CD44 knockout eliminates the ability of C1q to promote chemotaxis, but not the ability to promote division, in NSCs. Likewise, the effects of C1q on NSC differentiation appear to be independent of signaling via CD44. Nonetheless, C1q also exhibited a dose-dependent effect on NSC differentiation: while a low nM dose significantly increased the generation of Olig-2+ oligodendrocyte-lineage cells in wild-type NSCs, fewer Olig-2+ cells were seen with higher concentrations of C1q in both wild-type and CD44 knock-out cells. A similar inhibitory effect was observed on the differentiation of two other cell types (GFAP+ astroglia and ßIII-tubulin expressing neuronal lineage cells), which also appeared to be independent of signaling via CD44.

Benavente et al. also demonstrate that C1q, again acting via CD44, has important effects on the ability of transplanted NSCs to promote recovery from spinal cord injury. Previously they had shown that delayed transplantation into a rodent model of spinal cord injury was associated with the migration of cells away from the injury epicenter, differentiation of the NSCs mostly into oligodendrocytes, and improved motor function (Salazar et al., 2010; Hooshmand et al., 2017). In contrast, transplantation right after the injury was associated with increased migration towards the epicenter, differentiation mostly into astroglial cells, and no effects on motor recovery. Now, they have found that the genetic inhibition of CD44 in NSCs, or the use of antibodies to inhibit C1q, prevented clustering of transplanted NSCs in the injury epicenter, reduced astroglial differentiation, and significantly improved motor outcomes when NSCs were transplanted into mice immediately after injury. They also found elevated levels of C1q in the injury epicenter following spinal cord injury, which suggests that this pathway has a role in the response to the injury.

The latest work also raises a number of interesting questions. Would inhibiting inflammation increase the utility of NSC transplantation in spinal cord injury? Does the efficacy of delayed transplantation depend upon decreased inflammation and C1q production in the injury site by this time? Moreover, the binding of C1q to other NSC receptor proteins (including two G-protein coupled receptors and the receptor tyrosine kinase c-Met) suggests that C1q may modulate a complex array of effects on NSC behavior within the injury site.

One would also like to know if the latest results are relevant to recovery in areas of the central nervous system where NSCs still exist in adults, and whether there are effects of age-related increases in C1q on these NSC compartments. Moreover, CD44 is expressed in many different cell types, and its expression changes dramatically during injury: are other cells susceptible to C1q-mediated effects on chemotaxis via CD44? Also, if the normal expression of C1q in an injury site is to promote the migration of cells that might be important in repair, would decreasing the inflammatory response be counterproductive in some instances? It would also be interesting to explore the effects of C1q on cell function more generally.

The observations that C1q is a ligand for multiple receptors may also lead to a better understanding of the evolution of the complement system. The evolutionary value of a single protein that works as a component of a multi-protein complex is difficult to discern. However, if C1q started off as a signaling ligand, its evolution is easier to understand. In addition, one wonders whether other components of the complement cascade might also have additional roles outside of their immunological functions.

Regardless of the answers to these questions, the discovery that C1q is itself a ligand for multiple receptors, and is capable of modulating important NSC functions, opens up a new window for studying the biology of C1q that is sure to lead to a series of provocative further discoveries.
